# The Impact of Dietary Carbohydrates on Inflammation-Related Cardiovascular Disease Risk: The ATTICA Study (2002–2022)

**DOI:** 10.3390/nu16132051

**Published:** 2024-06-27

**Authors:** Sofia-Panagiota Giannakopoulou, Smaragdi Antonopoulou, Christina Chrysohoou, Fotios Barkas, Costas Tsioufis, Christos Pitsavos, Evangelos Liberopoulos, Petros P. Sfikakis, Demosthenes Panagiotakos

**Affiliations:** 1 Department of Nutrition and Dietetics, School of Health Sciences and Education, Harokopio University, 17676 Athens, Greece; 2 First Cardiology Clinic, Medical School, National and Kapodistrian University of Athens, Hippokration Hospital, 15772 Athens, Greece; 3 Department of Internal Medicine, Medical School, University of Ioannina, 45500 Ioannina, Greece; 4 First Department of Propaedeutic Internal Medicine, Medical School, National and Kapodistrian University of Athens, Laiko General Hospital, 15772 Athens, Greece

**Keywords:** cardiovascular disease, risk, carbohydrates, fiber, inflammatory biomarkers

## Abstract

The aim of this study was to evaluate the potential interplay between a carbohydrate diet and inflammation in atherosclerotic cardiovascular disease (ASCVD) development. ATTICA is a prospective observational study of 3042 adults free of cardiovascular disease (CVD) who were recruited in 2002 and followed for 20 years. Baseline data on carbohydrate intake and inflammatory biomarker levels were collected. Participants were stratified by carbohydrate intake (low vs. high: </> 190 g/day) and carbohydrate quality. At the 20-year follow-up in 2022, 1988 participants had complete data for CVD assessment. The overall quantity and quality of carbohydrate intake did not show a significant association with CVD incidence; inflammatory markers were positively correlated with an increased risk of CVD (*p*-values < 0.05). Chronic systemic inflammation seems to affect the CVD risk of participants who had a higher carbohydrate intake more substantially, as compared to those with low intake. Additionally, individuals with higher high carbohydrate/low fiber intake experienced a higher risk of inflammation-related CVD, compared to those with high carbohydrate/high fiber intake. The presented findings revealed that the effect of inflammation markers on the CVD risk is influenced both by the amount and quality of carbohydrate intake, irrespective of overall dietary habits and clinical and lifestyle characteristics.

## 1. Introduction

Carbohydrates, a major source of dietary energy [1], have been the focus of increasing attention in recent years, especially in the context of the obesity epidemic. However, their role in cardiovascular disease (CVD) remains under debate, as some studies suggest a link between carbohydrate intake and CVD risk and others show no clear connection [2].

Chronic inflammation is a key player in the development of atherosclerosis. High levels of inflammatory markers like C-reactive protein (CRP), interleukin-6 (IL-6), and tumour necrosis factor-alpha (TNF-α) are strong predictors of CVD risk and are also associated with insulin resistance and metabolic syndrome [3]. CRP is a key indicator of systemic inflammation and a predictor of cardiovascular events and is closely linked to visceral adiposity, driven by adipocyte-mediated cytokines [4]. Similarly, TNF-α, a multifunctional pro-inflammatory cytokine produced by adipose tissue, and IL-6, another major pro-inflammatory marker, are linked to various health implications including insulin resistance, neurodegeneration, and cancer [5,6].

The influence of carbohydrates on inflammatory processes remains an area of debate. While their role in insulin secretion and fat storage is undeniable [7], a definitive link to direct inflammatory effects is lacking. Recent studies suggest that carbohydrates might contribute to inflammation [8]. Further support comes from research indicating that low-carbohydrate diets may be associated with greater reductions in inflammatory markers compared to low-fat diets [9]. Additionally, the quality of carbohydrates is an important factor in nutrition, affecting health outcomes and overall well-being. For example, carbohydrate diets, including low glycemic index foods, that are rich in fiber have been shown to improve digestion, promote saturation, and help regulate blood glucose levels [10]. 

Traditionally, reducing saturated fat intake for lower low-density lipoprotein (LDL) cholesterol levels has been a focus of CVD prevention. However, it is crucial to consider macronutrient replacement. Replacing saturated fat with refined carbohydrates may increase CVD risk via elevated triglycerides and small LDL particles [11]. However, the interactions between carbohydrate intake and inflammatory markers remains understudied. Based on the aforementioned considerations, this study aims to investigate the role of carbohydrate intake (quantity and quality expressed as fiber content) on the risk of developing CVD events through its influence on chronic systemic inflammation process. The research hypothesis is that both quantity and quality of carbohydrate intake triggers inflammation process, leading to the development of CVD.

## 2. Materials and Methods

### 2.1. Study Design

The ATTICA study is a prospective (2002–2022) epidemiological cohort study with three follow-up assessments (in 2006, 2012, and 2022). The study’s objectives were to record the distribution of various socio-demographic, lifestyle, clinical, biochemical, and psychological risk factors for CVD; to explore the correlation between these factors and long-term CVD risk; and to evaluate their predictive value for CVD.

### 2.2. Setting and Participants

The study recruited a representative sample of 3042 inhabitants (4056 were invited, i.e., 75% participation rate) from the Attica region of Greece. The sample selection process ensured balanced representation by stratifying participants based on sex, age group, and region, reflecting the demographics of the 2001 census. All participants were free of pre-existing cardiovascular disease (CVD), cancer, and chronic inflammatory conditions at baseline.

More details about the study’s objectives, design, sampling method, and methodology are available in previously published papers [12,13,14].

### 2.3. Endpoint and Follow-Up Examination

In 2022, from the initial 3042 participants, 2169 were located and agreed and gave their written consent to participate in the follow-up study (participation rate: 71%). Among the 873 individuals who were not followed up on, 771 were unreachable due to changes in their contact details, while 102 opted out of the screening. 

In this study, the primary focus was on the incidence of fatal or non-fatal CVD events, as classified by the World Health Organization (WHO)—International Coding Diseases (ICD)-10 standards [12]. Data for participants who passed away were collected from relatives and death certificates.

This study encompassed 1988 participants who had comprehensive CVD data at all follow-up evaluations. The comparison of the age, sex, inflammatory markers, and carbohydrate consumption distribution of this subset with the initial group revealed no significant disparities (*p*-values > 0.05).

### 2.4. Baseline Assessment

Each participant underwent a comprehensive baseline assessment conducted by trained physicians. This assessment included a thorough physical examination, along with the compilation of a detailed medical history. Medical history included information regarding existing cardiovascular risk factors, current medications relevant to cardiovascular health, and any family history of cardiometabolic diseases. Additionally, the baseline assessment included a broad range of measurements, i.e., sociodemographic characteristics, anthropometric data, lifestyle habits, clinical evaluations, and biochemical measurements.

#### 2.4.1. Socio-Demographic Characteristics and Lifestyle

In this study, the sociodemographic factors evaluated were age (expressed in years) and sex (categorized as men and women). 

#### 2.4.2. Lifestyle Characteristics

Participants’ physical activity levels were assessed using the validated Greek version of the short International Physical Activity Questionnaire (IPAQ) [15]. Comparisons were drawn between inactive and active individuals, with activity levels quantified in terms of MET—minutes per week [16]. The participants’ smoking status was recorded. Additionally, the cumulative smoking exposure, expressed in pack years, was computed.

#### 2.4.3. Anthropometric Measurements

Standard procedures were used to measure body weight and height, waist, and hip circumference, and to calculate body mass index (BMI).

#### 2.4.4. Dietary Ascertainment 

The study employed the EPIC-Greek questionnaire, a validated 156-item semi-quantitative food frequency questionnaire (FFQ) specifically designed for the Greek population [17], to assess dietary habits at baseline and follow-up examinations. Trained dietitians assisted participants in completing the questionnaire, ensuring accuracy. Participants reported their average intake of each food item over the past year, using photographs as portion size guides. This comprehensive approach allowed researchers to capture habitual intake of individual foods and food groups.

Food composition tables were used to convert reported food intake into macronutrient intake (carbohydrates, protein, fat, dietary fiber). Energy intake was then calculated by summing the energy contributions from each macronutrient and alcohol, using established conversion factors (4 kcal/g for carbohydrates and protein, 9 kcal/g for fat, and 7 kcal/g for alcohol). This allowed the estimation of daily energy intake (in kcal) and total carbohydrate intake (in grams per day, g/day), along with the percentage of energy derived from carbohydrates. 

Carbohydrate quality was assessed using a combined approach considering both carbohydrate and fiber content, retrieved from food composition tables. Median values were computed for both total carbohydrates (11.85 g/100 g) and dietary fiber (2.45 g/100 g). Foods were categorized as “High Carbohydrate/High Fiber” if both their carbohydrate and fiber content exceeded the respective medians, and as “High Carbohydrate/Low Fiber” if their carbohydrate content was above the median but their fiber content was below it. To assess overall carbohydrate quality, two composite scores were created by summing the intake of foods in each category. These scores were then combined to form a single binary variable representing carbohydrate quality. The low quantile indicated a dietary pattern characterized by higher consumption of “High Carbohydrate/Low Fiber” foods or lower intake of “High Carbohydrate/High Fiber” foods, representing lower carbohydrate quality. Conversely, the high quantile indicated a dietary pattern with lower consumption of “High Carbohydrate/Low Fiber” foods or higher intake of “High Carbohydrate/High Fiber” foods, indicating higher carbohydrate quality.

The MedDietScore, which has a range from 0 to 55, was used to assess overall adherence to the Mediterranean dietary pattern [18].

#### 2.4.5. Biochemical Measurements and Clinical Characteristics

Blood samples were collected in a fasting state between 08:00 and 10:00 h. The biochemical analysis was performed in a laboratory that adheres to the standards of the World Health Organization Reference Laboratories. High-sensitivity C-reactive protein was tested using particle-enhanced immunonephelometry. Interleukin-6 was quantified using a high-sensitivity enzyme-linked immunoassay. The intra-assay and inter-assay variability was less than 5% for C-reactive protein and less than 10% for interleukin-6. The ELISA method was employed for the quantitative measurement of human tumor necrosis factor-α in duplicate in the participants’ serum samples, using the Quantikine HS/human tumor necrosis factor-α immunoassay kit.

Standardized criteria to define the specific clinical characteristics of the participants were employed. In terms of clinical characteristics, participants were considered to have hypercholesterolemia if their total cholesterol exceeded 200 mg/dL or they were on lipid-lowering medications. Similarly, hypertension was defined based on either a systolic blood pressure (SBP) greater than 140 mmHg or a diastolic blood pressure (DBP) exceeding 90 mmHg, or the use of antihypertensive medications. Finally, type 2 diabetes mellitus (hereafter referred to as diabetes) was defined by a fasting blood glucose level above 126 mg/dL or the current use of insulin and/or oral hypoglycemic medications.

### 2.5. Follow-Up Assessment

Thorough clinical information was gathered through in-person interviews conducted by experienced healthcare professionals and from medical records. Every participant was reached out to via phone, evaluated, and asked to participate in the follow-up. In cases where participants had passed away during the follow-up, details were obtained from their family members and official death certificates. The vital status of participants, including deaths, causes of death, and non-fatal CVD events, was also recorded following the World Health Organization (WHO)—International Coding Diseases (ICD)-10 classification [12].

### 2.6. Statistical Analysis

Continuous variables were expressed as mean values ± standard deviation, while categorical variables were expressed as frequencies. The chi-squared test was used to examine the associations between categorical variables. Continuous variables were tested for normality using P–P plots. The *t*-test was used to compare the mean values of normally distributed variables between those who experienced an event and the remaining participants, after ensuring variance equality with Levene’s test. Group comparisons were conducted using either the Welch’s *t*-test for normally distributed continuous variables with potentially unequal variances, or the Mann–Whitney U test for non-normally distributed continuous variables. The crude incidence of CVD was calculated by dividing the number of new cases by the total number of participants in the 20-year follow-up study. Participants were categorized (a) into two groups based on their carbohydrate intake using a median split, resulting in high and low carbohydrate intake groups, and (b) into two groups based on their carbohydrate quality, i.e., high carbohydrate/low fiber and high carbohydrate/high fiber. Cox proportional hazard models were used to investigate the relationship between baseline inflammatory markers levels and the risk of developing CVD over 20 years. The results are presented as hazard ratios (HR) with 95% confidence intervals (CI). Stratification by carbohydrate intake and dietary pattern was used to explore potential interaction between carbohydrate consumption and inflammatory markers. To examine the moderating effects of other CVD risk factors on the relationship between inflammatory markers and 20-year incidence of CVD, separate models were estimated for each potential moderator, stratified by carbohydrate intake. Forest plots were then generated to visually represent these interactions. All reported *p*-values were based on two-tailed hypotheses and compared to a level of 5% for determining significance. STATA version 18 (STATA Corp, College Station, TX, USA) was used for the statistical analyses.

## 3. Results

### 3.1. CVD Incidence and Mortality at 20-Year Follow-Up

**Figure 1:**
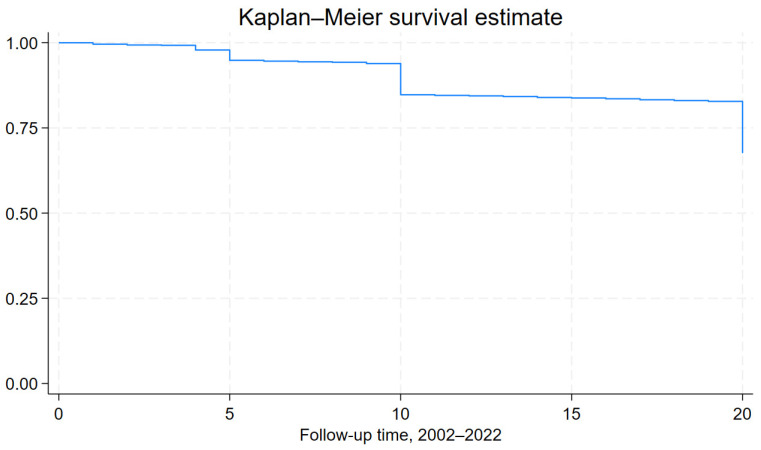
Kaplan–Meier survival curve for the ATTICA study sample, during the 20-year follow-up period, 2002–2022.

Over a 20-year period, 718 (36 per 100) participants experienced a fatal or non-fatal CVD event (40 per 100 for males and 32 per 100 for females, *p* for sex differences < 0.001). These events included coronary heart disease (CHD) (71.7%), stroke (4.3%), and other types of events (i.e., heart failure, peripheral arterial disease, and aortic disease) (24%). Among the 718 CVD incidents, 96 were fatal, resulting in a 20-year CVD mortality rate of 7.3% in men and 1.8% in women (*p* < 0.001). Figure 1 illustrates the Kaplan–Meier survival rate of the study’s participants throughout the 20-year follow-up period. 

Data presented in Table 1 demonstrate a robust association between the majority of established lifestyle and clinical CVD determinants and the observed long-term incidence of CVD within the study cohort. Similarly, inflammatory markers were significantly higher in participants who experienced CVD events during the follow-up period, compared to those who remained free of CVD. In general, those who developed CVD over the 20 years course were predominantly older males with elevated inflammatory markers. They also had a higher prevalence of obesity and a history of hypertension, hypercholesterolemia, and diabetes mellitus at baseline, compared to those who remained free of CVD.

### 3.2. Participants’ Characteristics by Carbohydrate Intake and Carbohydrate Quality

In Table 2, the characteristics of the participants based on their carbohydrate intake (low, <190 g/day; high, >190 g/day) are presented. It was observed that those who adhered to a high carbohydrate diet were mainly males, of younger age, and whose consumption was closer to the Mediterranean dietary pattern, compared to those who followed a low carbohydrate intake diet. No other significant differences regarding lifestyle, clinical characteristics, or inflammatory marker levels were observed. Similarly, participant categorization by carbohydrate quality (Table 3) revealed no significant differences in lifestyle factors, clinical characteristics, or inflammatory marker levels, with the exception of baseline hypertension.

No association was observed between carbohydrate intake and risk of developing CVD events during the 20-year period in the age–sex adjusted model (HR per 100 g/day = 0.944, 95% CI 0.725–1.231), as well as in the full model after adjusting for MedDietScore, physical activity levels, smoking habits, BMI, total energy intake as well as medical history of hypertension, diabetes, hypercholesterolaemia, and family history of CVD (HR per 100 g/day = 0.612; 95% CI 0.282–1.329). Similarly, this study investigated the potential relationship between carbohydrate quality and CVD risk. Again, no statistically significant association was found between carbohydrate quality and CVD risk in either the age- and sex-adjusted model or the fully adjusted model incorporating the covariates (age–sex adjusted model HR = 0.935, 95% CI 0.517–1.694; fully adjusted model HR = 1.022, 95% CI 0.524–1.996).

### 3.3. Inflammation Indices and 20-Year CVD Incidence

A significant interaction was observed between carbohydrate parameters (intake and quality) and inflammatory markers levels on CVD risk (*p*-values < 0.10). Table 4 and Table 5 present the results of nested survival models examining the association between baseline inflammatory markers and the 20-year incidence of CVD, stratified by carbohydrate intake and carbohydrate quality, respectively.

In the unadjusted models for the entire cohort, a significant association between the baseline hs-CRP, IL-6, and TNF-α and the 20-year incidence of CVD is observed. However, these associations were no longer significant after adjusting for demographic, lifestyle, and clinical factors (i.e., age, sex, BMI, smoking status, physical activity level, adherence to a Mediterranean diet, hypercholesterolemia, hypertension, diabetes, and family history of CVD).

While neither carbohydrate intake nor carbohydrate quality directly affected CVD risk, they significantly modulated the influence of inflammatory markers on CVD risk (interaction *p*-values < 0.10). Stratification by carbohydrate intake revealed a more pronounced influence of TNF-α on CVD risk within the high-carbohydrate consumption group (HR per 0.1 pg/mL increase: high vs. low carbohydrate: 1.024 (95% CI 1.014–1.035) vs. 1.012 (95% CI 1.004–1.019); *p* for interaction < 0.1). In the fully adjusted model, similar results were observed for hs-CRP as stratification by carbohydrate intake levels revealed a stronger effect of hs-CRP on CVD risk among participants with higher carbohydrate intake (HR per 1 mg/L increase: high vs. low carbohydrate: 1.160 (95% CI 1.004–1.341) vs. 0.886 (95% CI 0.735–1.068); *p* for interaction < 0.1). Similarly, when stratified by carbohydrate quality, a more prominent association between all examined inflammatory markers and the risk of CVD was revealed among individuals in the high carbohydrate/low fiber group. In the unadjusted model, the impact of all the investigated inflammation markers on CVD risk was notably stronger in the high carbohydrate/low fiber group (*p* for interaction < 0.1). In the fully adjusted model, similar trends were observed for hs-CRP and IL-6, indicating an increased effect of these markers on CVD risk among those in the high carbohydrate/low fiber group (*p* for interaction < 0.1). 

### 3.4. Moderation Analyses 

**Figure 2:**
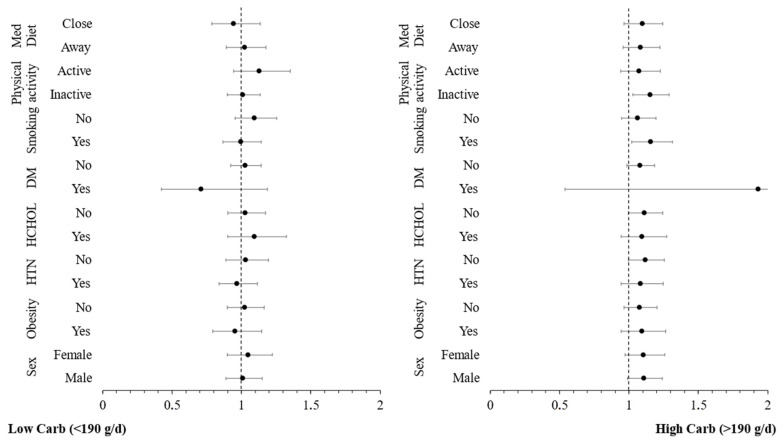
Plot of hazard ratios and 95% CIs of high sensitivity C-reactive protein (hs-CRP) in relation to 20-year cardiovascular disease (CVD) incidence across different subgroups of participants from the ATTICA study, categorized by carbohydrate intake (HTN, presence of hypertension; HCHOL; presence of hypercholesterolemia; DM, diabetes mellitus; Med Diet, Mediterranean diet).

**Figure 3:**
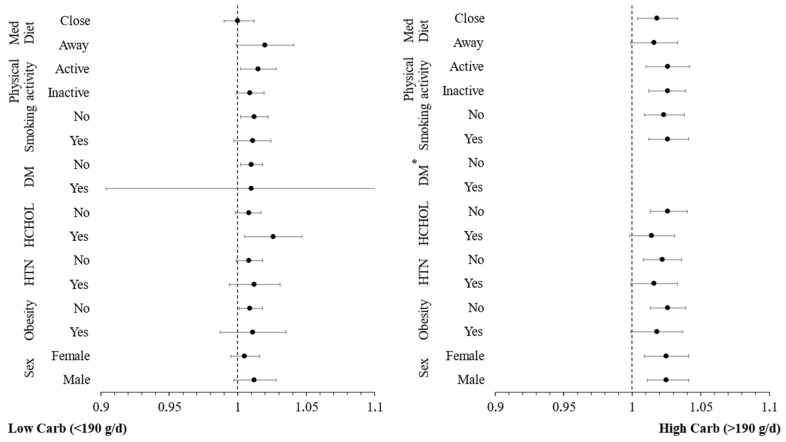
Plot of hazard ratios and 95% CIs of tumor necrosis factor-alpha (TNF-α) in relation to 20-year cardiovascular disease (CVD) incidence across different subgroups of participants from the ATTICA study, categorized by carbohydrate intake (HTN, presence of hypertension; HCHOL; presence of hypercholesterolemia; DM, diabetes mellitus; Med Diet, Mediterranean diet). * Redundant for DM.

**Figure 4:**
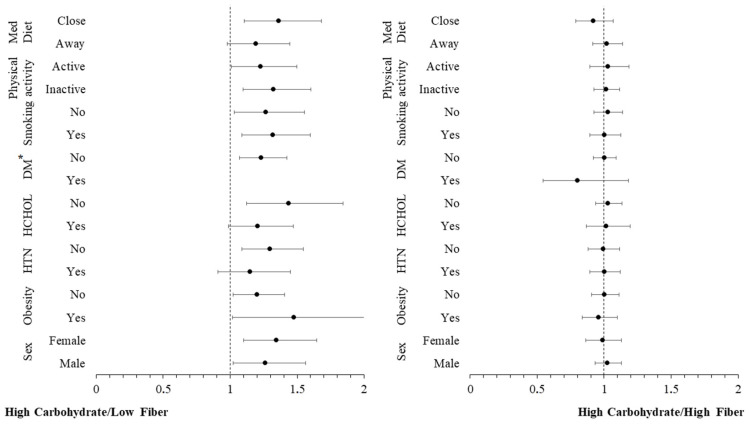
Plot of hazard ratios and 95% CIs of high sensitivity C-reactive protein (hs-CRP) in relation to 20-year cardiovascular disease (CVD) incidence across different subgroups of participants from the ATTICA study, categorized by carbohydrate quality (HTN, presence of hypertension; HCHOL; presence of hypercholesterolemia; DM, diabetes mellitus; Med Diet, Mediterranean diet). * Redundant for DM.

**Figure 5:**
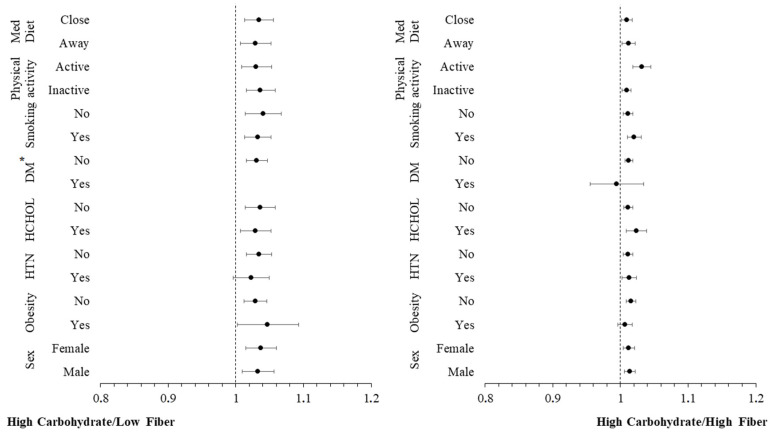
Plot of hazard ratios and 95% CIs of interleukin-6 (IL-6) in relation to 20-year cardiovascular disease (CVD) incidence across different subgroups of participants from the ATTICA Study, categorized by carbohydrate quality (HTN, presence of hypertension; HCHOL; presence of hypercholesterolemia; DM, diabetes mellitus; Med Diet, mediterranean diet). * Redundant for DM.

**Figure 6:**
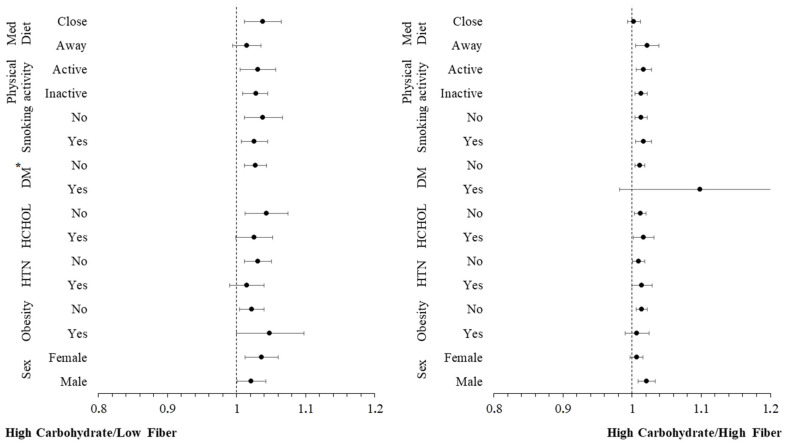
Plot of hazard ratios and 95% CIs of tumor necrosis factor-alpha (TNF-α) in relation to 20-year cardiovascular disease (CVD) incidence across different subgroups of participants from the ATTICA study, categorized by carbohydrate quality (HTN, presence of hypertension; HCHOL; presence of hypercholesterolemia; DM, diabetes mellitus; Med Diet, Mediterranean diet). * Redundant for DM.

Additional moderation analyses were conducted to investigate whether other CVD risk factors (sex, obesity, hypertension, hypercholesterolaemia, diabetes, smoking, physical inactivity, low adherence to Mediterranean diet) have an additional impact on the moderating role of carbohydrate intake in the relationship between inflammatory markers and incidence of CVD. The hazard ratios for the incidence of CVD over a 20-year period, across these CVD risk factors, are graphically presented in Figure 2 and Figure 3. These figures are categorized by carbohydrate intake and are based on concentrations of hs-CRP and TNF-α, respectively. The analysis revealed no significant interaction effect between hs-CRP and TNF-α levels and these CVD risk factors (all *p*-values > 0.10), indicating that the influence of these predictors on the CVD outcome is consistent, irrespective of the levels of the potential moderating factors. Stratifying by carbohydrate quality, the moderation analysis revealed no significant interaction effect between hs-CRP (Figure 4), IL-6 (Figure 5) and TNF-α (Figure 6) levels and these CVD risk factors (all *p*-values > 0.05), with the exception of physical activity which appeared to moderate the association between IL-6 and CVD for individuals following a high carbohydrate–high fiber diet.

## 4. Discussion

This study provides, to the best of our knowledge, new evidence on the potential moderating role of carbohydrate intake on the association between inflammation and CVD risk. Chronic, low-grade inflammation is intimately linked to the development of CVD [19], as it triggers the early phases of the atherogenesis [20]. Among pro-inflammatory dietary patterns, Western-style diets—high in refined carbohydrates and low in fiber—have raised concern as potential modulators of chronic systemic inflammation [21,22]. The presented findings revealed that the effect of inflammation markers on the CVD risk depends on the level of both carbohydrate intake and quality, suggesting that high carbohydrate–low fiber intake triggers the effect of inflammation process on atherosclerotic cardiovascular disease (ASCVD) risk. The public health consequence of the presented findings is significant, as they indicate that dietary choices, specifically the intake and quality of carbohydrates, have a crucial impact on the relationship between inflammation markers and cardiovascular risk. A diet high in carbohydrates but low in fiber can exacerbate inflammation processes, thereby increasing the risk of ASCVD.

Refined carbohydrate consumption is implicated in promoting chronic low-grade inflammation through various mechanisms. High carbohydrate intake, especially refined carbs, contributes to hyperglycemia and hyperinsulinemia [23,24]. Hyperglycemia-induced oxidative stress is a key factor contributing to chronic low-grade inflammation [25], as promotes a pro-inflammatory and oxidative stress environment through the generation of reactive oxygen species (ROS) [26]. These ROS cause the oxidative modification of LDL-cholesterol [27] and, in early phases, activate NF-κB [28], a regulator of apoptosis and pro-inflammatory cytokine expression [29]. This activation cascade leads to the upregulation and release of pro-inflammatory cytokines, such as TNF-α and IL-6 [29], which in turn stimulate CRP expression [30], a marker of vascular inflammation and a significant predictor of CVD risk. Additionally, TNF-α induces the expression of adhesion molecules like vascular cell adhesion molecule-1 (VCAM-1) and intercellular adhesion molecule-1 (ICAM-1) on endothelial cells, known to be involved in atherogenesis [21]. Another pathway through which refined carbohydrate consumption affects inflammation is via the gut microbiota, where high-sugar diets lead to the increased production of bacterial pro-inflammatory factors like lipopolysaccharides (LPS). This diet-induced gut inflammation impairs the epithelial barrier, facilitating increased LPS uptake into the circulation (metabolic endotoxemia) and consequently triggering systemic inflammation [31]. Additionally, exogenous (dietary) AGEs (dAGEs) intake from the consumption of foods high in sugar or foods exposed to high-temperature cooking methods, accelerates the production of endogenous AGEs. These endogenous AGEs, acting through receptor and non-receptor mechanisms, activate different cells, increasing oxidative stress and the release of pro-inflammatory cytokines [22,32].

In the present study, it was observed that carbohydrate intake is not directly associated with the risk of CVD, an observation consistent with some [33,34], but not all, studies, as some research reports a positive association [2,35]. In examining carbohydrate intake and its impact on cardiometabolic risk, a critical factor beyond just quantity is carbohydrate quality [2,36,37], which was evaluated in the present study. Emphasis was placed on fiber content as a measure of carbohydrate quality. Although the glycemic index, glycemic load, and specific fiber sources have been investigated as indicators of quality, their applicability is limited due to inconsistent findings or a lack of sufficient data to definitively categorize carbohydrates as low or very low quality [1]. Higher intakes of total dietary fiber and whole grains, both contributors to total carbohydrate intake, are associated with a reduced risk of CVD [37,38,39,40]. Conversely, diets high in carbohydrates with a high glycemic index are linked to an increased risk of CVD [41]. Soluble and viscous fiber types exert beneficial effects via many potential mechanisms. These fibers form gels within the small intestine which influence absorption and consequently attenuate postprandial elevations in blood glucose and lipids. Additionally, gel formation promotes satiety by delaying gastric emptying, potentially contributing to weight management. Furthermore, soluble fiber and resistant starch undergo fermentation by gut bacteria, yielding short-chain fatty acids that assist in reducing circulating cholesterol levels [40,42].

Our findings strongly suggest a potential interaction between carbohydrate intake and chronic systemic inflammation markers in influencing CVD risk, as inflammation seems to affect more substantially CVD risk among participants who had a higher intake of carbohydrates as compared to those with a low intake, irrespective of overall dietary habits. Εach 1 mg/L increase in hs-CRP levels resulted in a 16% greater risk of developing CVD for participants with high carbohydrate intake compared to those with lower carbohydrate intake, irrespective of overall dietary habits. This aligns with findings from other studies that demonstrate reductions in inflammatory markers associated with low-carbohydrate diets, even without substantial calorie restriction or weight loss [43,44]. Moderation analysis revealed that there was no significant interaction between hs-CRP levels in relation to the studied CVD risk factors, irrespective of carbohydrate intake, thus indicating that the influence of hs-CRP on the CVD outcome is consistent.

Our study also strongly suggests an interaction between carbohydrate quality and chronic systemic inflammation markers in influencing CVD risk. Specifically, participants following a high carbohydrate-low fiber diet exhibited a more pronounced impact of inflammation on CVD risk. Within this dietary group, a 1 mg/L increase in hs-CRP levels translated to a 40.5% greater risk of developing CVD, while a 0.01 pg/mL increase in IL-6 levels resulted in a 3.4% increased risk. These associations remained significant even after adjusting for demographic characteristics, lifestyle habits, and clinical factors, suggesting that a high-carbohydrate, low-fiber dietary pattern may accentuate the adverse effects of chronic inflammation on cardiovascular health. This is consistent with results from other research that show a correlation between fiber consumption and inflammation [45,46]. Moderation analysis revealed that there was no significant interaction between these inflammatory biomarker levels in relation to the studied CVD risk factors, irrespective of carbohydrate quality, thus indicating that the influence of these biomarkers on the CVD outcome is consistent. The one exception was physical activity, which appeared to moderate the association between IL-6 and CVD for individuals following a high carbohydrate-high fiber diet. Notably, working muscles acutely produce IL-6, and the extent of this response depends on exercise intensity, duration, and muscle glycogen levels [47]. Therefore, it is possible this interaction might depend on the type, intensity, and duration of physical activity, which were not evaluated in the present study.

There is no standard definition for a “low-carbohydrate” diet that has been universally accepted among the scientific community. The acceptable macronutrient distribution range (AMDR) for carbohydrates is 45−65% of daily caloric intake, with a recommended dietary allowance (RDA) of 130 g of carbohydrate per day across all age and sex groups, mainly based on the average amount of glucose utilized daily by the brain [48]. However, European Food Safety Authority (EFSA) labeling guidelines suggested a higher reference intake (260 g/day) which fall within the lower limits of recommended intakes for the general population and approached the upper end of average carbohydrate intakes in adults across EU countries [49]. This carbohydrate consumption is aligned with observed consumption patterns, where median intakes ranging from 180–230 g/day for women and 200–330 g/day for men [50]. While a universally accepted definition for “low-carbohydrate” is lacking, our study using a 190 g/day threshold (corresponding to 36.6% of daily calories) demonstrates a potential link between higher carbohydrate consumption and increased CVD risk. This suggests that a threshold may exist for carbohydrate intake beyond which inflammation-driven CVD risk increases. However, establishing a definitive threshold to mitigate inflammation-related CVD risk requires further investigation. Future research should focus on determining the optimal level of carbohydrate intake that promotes cardiovascular health, considering individual factors and overall dietary composition.

### Strengths and Limitations

This study has several strengths. To the best of our knowledge, the ATTICA study is the sole large-scale, prospective cohort study examining CVD epidemiology within the Greek population. Furthermore, it stands out from other studies due to its extensive follow-up period with multiple data collection waves. The study’s sample was both sufficient and representative in mirroring the age and sex distribution of the urban Greek population. A comprehensive evaluation of participants was carried out at the baseline in terms of clinical, biochemical, and lifestyle factors related to cardiovascular disease. The study examined the impact of carbohydrate intake and quality on inflammation-related CVD risk, while accounting for various risk factors and investigating the potential moderating effects. To the best of our knowledge, there appears to be insufficient data on this subject.

This study has also some limitations. It only considered baseline measurements, which could result in inaccuracies in tracking changes because of the long interim periods between follow-up evaluations. The lack of any information regarding data regarding CVD for participants lost to follow-up limits the generalizability of our findings. The research focused on a group with low cardiovascular disease risk, i.e., individuals without existing heart disease. Finally, isolating the specific effects of individual macronutrients on health outcomes is challenging due to their interdependence. Adjusting the intake of one macronutrient, such as carbohydrate, necessarily necessitates adjustments to fat and protein intakes in order to maintain total energy intake. This complexity makes it difficult to definitively attribute specific health outcomes solely to carbohydrate intake.

## 5. Conclusions

The relationship between carbohydrates and CVD is complex. While overall carbohydrate consumption and quality may not directly trigger CVD, our study revealed that a low-fiber high-carbohydrate diet could potentiate the effect of specific inflammatory markers on CVD risk. This interplay underscores the importance of personalized dietary recommendations to prevent CVD, particularly for individuals with chronic systemic inflammation.

## Figures and Tables

**Table 1:** Characteristics of the ATTICA study’s participants (n = 1988) according to the 20-year incidence of CVD.

	Status at 20-Year Follow-Up			
	Overall (2002)	CVD Free (n = 1270)	CVD Event (n = 718)	*p* Value
	Demographic and lifestyle factors			
Age, mean ± SD	45 ± 14	38 ± 9	58 ± 11	<0.001
Male sex	50%	46%	55%	<0.001
Smoking Pack years, mean ± SD	497 ± 501	375 ± 340	703 ± 633	<0.001
Physical activity (2002–2012)				<0.001
Remained inactive	50%	46%	56%	
Remained active	13%	13%	14%	
Became inactive	28%	27%	29%	
Became active	10%	14%	2%	
MedDietScore (0–55), mean ± SD	26 ± 7	27 ± 6	23 ± 6	<0.001
Carbohydrate intake				
g/day	211 ± 97	215 ± 97	201 ± 91	0.088
% total energy intake	36.9 ± 6.5	36.8 ± 6.1	36.8 ± 6.9	0.946
	Clinical factors			
Obesity	18%	14%	27%	<0.001
Diabetes at baseline	7%	1%	17%	<0.001
Hypertension at baseline	30%	20%	51%	<0.001
Hypercholesterolemia at baseline	40%	30%	65%	<0.001
Family history of CVD	36%	36%	39%	0.210
	Inflammation indices			
hs-CRP (mg/L), mean ± SD	1.94 ± 2.42	1.78 ± 2.42	2.26 ± 2.44	<0.001
IL-6 (pg/mL), mean ± SD	1.46 ± 0.55	1.36 ± 0.46	1.63 ± 0.62	<0.001
TNF-α (pg/mL), mean ± SD	6.21 ± 4.90	5.63 ± 4.55	7.77 ± 5.03	<0.001

Abbreviations: hs-CRP: high sensitivity C-reactive protein; IL-6: interleukin-6; TNF-α: tumor necrosis factor-alpha.

**Table 2:** Characteristics of the ATTICA study’s participants according to carbohydrate intake.

	Carbohydrate Intake (g/day)		
	Low (<190 g/day)	High (>190 g/day)	*p* Value
	Demographic and lifestyle factors		
Age, mean ± SD	41 ± 11	39 ± 11	0.005
Male sex	49%	60%	<0.001
Smoking Pack years, mean ± SD	442 ± 412	418 ± 420	0.478
Physical activity (2002–2012)			0.155
Remained inactive	43%	39%	
Remained active	16%	22%	
Became inactive	27%	25%	
Became active	14%	14%	
MedDietScore (0–55), mean ± SD	26 ± 6	29 ± 10	<0.001
	Clinical factors		
Obesity	14%	18%	0.150
Diabetes at baseline	5%	4%	0.286
Hypertension at baseline	25%	29%	0.178
Hypercholesterolemia at baseline	33%	29%	0.223
Family history of CVD	36%	35%	0.955
	Inflammation indices		
hs-CRP (mg/L), mean ± SD	1.89 ± 2.39	2.03 ± 2.63	0.393
IL-6 (pg/mL), mean ± SD	1.42 ± 0.36	1.41 ± 0.36	0.778
TNF-α (pg/mL), mean ± SD	6.35 ± 3.18	6.31 ± 2.89	0.829

Abbreviations: hs-CRP: high sensitivity C-reactive protein; IL-6: interleukin-6; TNF-α: tumor necrosis factor-alpha.

**Table 3:** Characteristics of the ATTICA study’s participants according to carbohydrate quality.

	High Carbohydrate/Low Fiber	High Carbohydrate/High Fiber	*p* Value
	Demographic and lifestyle factors		
Age, mean ± SD	39 ± 11	40 ± 11	0.212
Male sex	54%	54%	0.972
Smoking Pack years, mean ± SD	435 ± 383	430 ± 423	0.906
Physical activity (2002–2012)			0.310
Remained inactive	47%	40%	
Remained active	20%	18%	
Became inactive	22%	27%	
Became active	12%	14%	
MedDietScore (0–55), mean ± SD	27 ± 8	27 ± 8	0.951
	Clinical factors		
Obesity	16%	16%	0.817
Diabetes at baseline	2%	5%	0.093
Hypertension at baseline	20%	29%	0.012
Hypercholesterolemia at baseline	36%	30%	0.117
Family history of CVD	30%	37%	0.085
	Inflammation indices		
hs-CRP (mg/L), mean ± SD	2.17 ± 2.86	1.92 ± 2.43	0.262
IL-6 (pg/mL), mean ± SD	1.43 ± 0.39	1.41 ± 0.35	0.640
TNF-α (pg/mL), mean ± SD	6.59 ± 3.30	6.27 ± 2.97	0.216

Abbreviations: hs-CRP: high sensitivity C-reactive protein; IL-6: interleukin-6; TNF-α: tumor necrosis factor-alpha.

**Table 4:** Results from nested Cox proportional hazards models exploring the association between baseline inflammation indices and the risk of developing a cardiovascular (CVD) event throughout the 20-year study period, according to carbohydrate intake. * *p* < 0.05.

	HR (95% CI) of CVD			
	Overall			
	Crude model	Model 1	Model 2	Model 3
hs-CRP (per 1 mg/L)	1.079 (1.038–1.122) *	1.053 (0.991–1.119)	1.049 (0.955–1.151)	1.051 (0.948–1.165)
IL-6 (per 0.01 pg/mL)	1.012 (1.009–1.014) *	1.000 (0.997–1.003)	1.000 (0.996–1.004)	1.006 (0.998–1.013)
TNF-α (per 0.1 pg/mL)	1.009 (1.007–1.012) *	1.001 (0.997–1.005)	1.003 (0.995–1.012)	1.001 (0.991–1.010)
		Low Carbohydrate intake (<190 g/day)		
	Crude model	Model 1	Model 2	Model 3
hs-CRP (per 1 mg/L)	1.045 (0.949–1.151)	0.996 (0.870–1.141)	0.919 (0.782–1.079)	0.886 (0.735–1.068)
IL-6 (per 0.01 pg/mL)	1.018 (1.010–1.026) *	1.005 (0.993–1.016)	0.999 (0.985–1.013)	0.998 (0.983–1.014)
TNF-α (per 0.1 pg/mL)	1.012 (1.004–1.019) *	0.996 (0.982–1.010)	0.987 (0.968–1.006)	0.983 (0.961–1.005)
	High Carbohydrate intake (>190 g/day)			
	Crude model	Model 1	Model 2	Model 3
hs-CRP (per 1 mg/L)	1.103 (1.014–1.199) *	1.127 (1.004–1.266) *	1.127 (0.990–1.284)	1.160 (1.004–1.341) *
IL-6 (per 0.01 pg/mL)	1.017 (1.010–1.024) *	1.007 (0.998–1.016)	1.007 (0.997–1.016)	1.010 (0.999–1.020)
TNF-α (per 0.1 pg/mL)	1.024 (1.014–1.035) *	1.012 (0.997–1.027)	1.014 (0.998–1.031)	1.014 (0.997–1.032)

Model 1: Age, sex. Model 2: Model 1 + MedDietScore, physical activity, smoking, BMI, total energy intake (kcal/d). Model 3: Model 2 + hypertension, diabetes, hypercholesterolaemia, family history of CVD. Abbreviations: hs-CRP: high sensitivity C-reactive protein; IL-6: interleukin-6; TNF-α: tumor necrosis factor-alpha.

**Table 5:** Results from nested Cox proportional hazards models exploring the association between baseline inflammation indices and the risk of developing a cardiovascular (CVD) event throughout the 20-year study period, according to carbohydrate quality. * *p* < 0.05.

	HR (95% CI) of CVD			
	High carbohydrate/Low fiber			
	Crude model	Model 1	Model 2	Model 3
hs-CRP (per 1 mg/L)	1.28 (1.11–1.47) *	1.25 (1.04–1.505) *	1.28 (1.03–1.60) *	1.40 (1.04–1.88) *
IL-6 (per 0.01 pg/mL)	1.03 (1.01–1.04) *	1.02 (1.005–1.040) *	1.03 (1.01–1.04) *	1.03 (1.00–1.06) *
TNF-α (per 0.1 pg/mL)	1.03 (1.01–1.04) *	1.02 (0.996–1.037)	1.02 (0.99–1.04)	1.02 (0.98–1.04)
	High carbohydrate/High fiber			
	Crude model	Model 1	Model 2	Model 3
hs-CRP (per 1 mg/L)	1.02 (0.94–1.09)	1.01 (0.90–1.12)	0.96 (0.85–1.09)	0.97 (0.84–1.11)
IL-6 (per 0.01 pg/mL)	1.01 (1.01–1.02) *	1.00 (0.99–1.01)	0.99 (0.99–1.01)	1.001 (0.99–1.01)
TNF-α (per 0.1 pg/mL)	1.01 (1.01–1.02) *	0.99 (0.98–1.01)	0.99 (0.98–1.01)	0.99 (0.98–1.01)
p for interaction				
	Crude model	Model 1	Model 2	Model 3
hs-CRP	0.004	0.052	0.045	0.095
IL-6	0.020	0.047	0.043	0.095
TNF-α	0.089	0.159	0.164	0.168

Model 1: Age, sex. Model 2: Model 1 + MedDietScore, physical activity, smoking, BMI, total energy intake (kcal/d). Model 3: Model 2 + hypertension, diabetes, hypercholesterolaemia, family history of CVD. Abbreviations: hs-CRP: high sensitivity C-reactive protein; IL-6: interleukin-6; TNF-α: tumor necrosis factor-alpha.

## Data Availability

The data presented in this study are available on request from the corresponding author. The data are not publicly available due to privacy restrictions.
